# Neutrophil Extracellular Traps Open the Pandora’s Box in Severe Malaria

**DOI:** 10.3389/fimmu.2017.00874

**Published:** 2017-07-28

**Authors:** Sebastian Boeltz, Luis E. Muñoz, Tobias A. Fuchs, Martin Herrmann

**Affiliations:** ^1^Department of Internal Medicine 3 – Rheumatology and Immunology, Friedrich-Alexander-University Erlangen-Nürnberg (FAU) and Universitätsklinikum Erlangen, Erlangen, Germany; ^2^Institute of Clinical Chemistry and Laboratory Medicine, University Medical Center Hamburg-Eppendorf, Hamburg, Germany

**Keywords:** severe malaria, cerebral malaria, neutrophil extracellular traps (NETs), Disseminated intravascular coagulation (DIC), vascular occlusion

## Introduction

**Figure 1:**
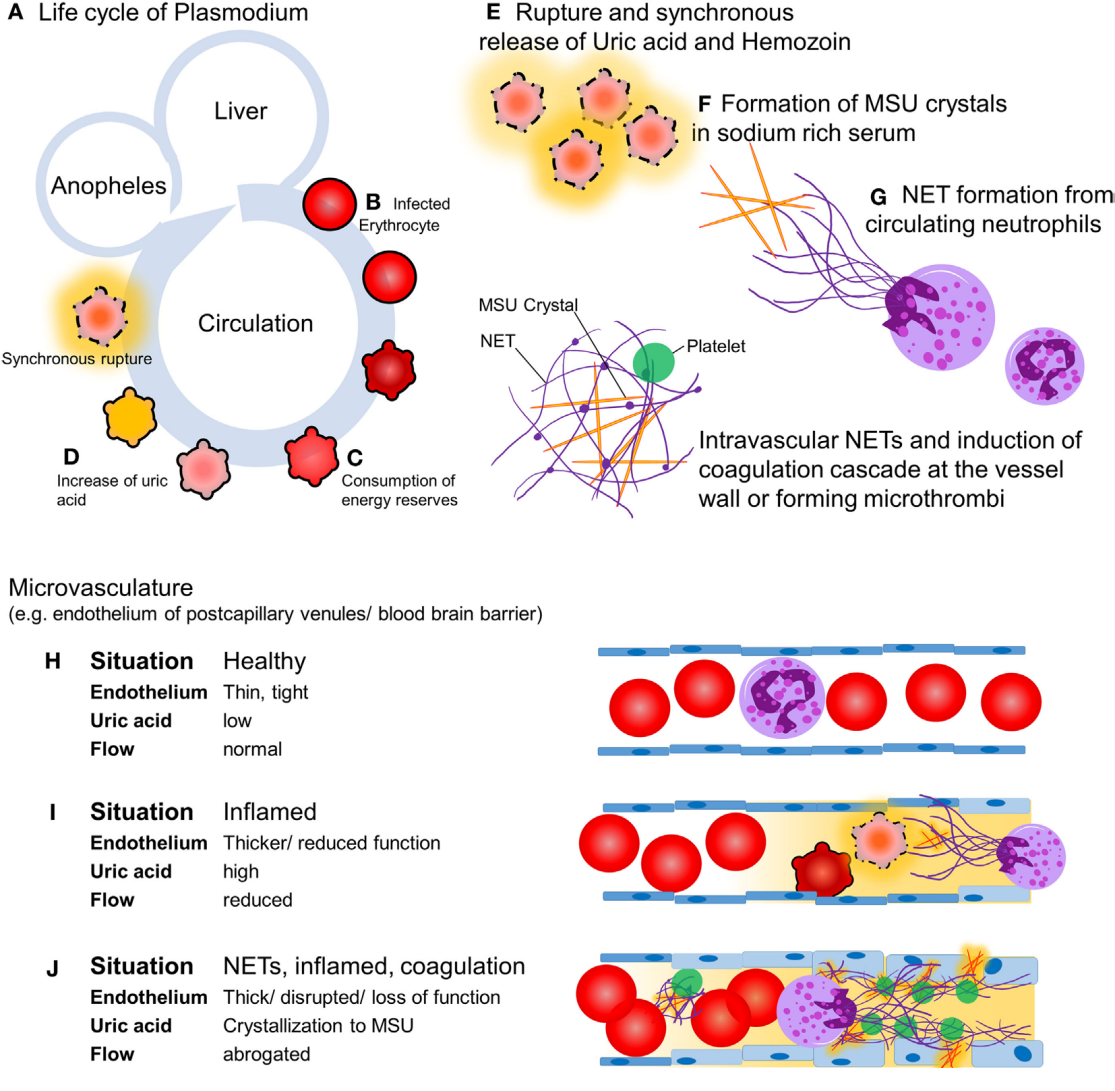
Hypothesis: intravascular neutrophil extracellular traps (NETs) precipitate severe malaria. Upon feeding, female *Anopheles* mosquitoes inoculate the host with sporozoites **(A)** which, within minutes, invade hepatocytes and replicate. A single sporozoite produces 10,000–30,000 merozoites within 5–8 days. After this the hepatocytes burst, merozoites enter the circulation, and invade erythrocytes and replicate **(B)**. Depending on the species, the blood stage takes 24, 48, or 72 h. During the growth within the erythrocytes, the parasite consumes the cells’ cytoplasm and energy resources **(C)**. Replicating parasites produce and store waste-like uric acid (orange) and hemozoin **(D)**. During the blood stage, the parasite population increases 6–20 times per cycle. If the resources of the erythrocyte are consumed, rupture of the host erythrocyte releases the parasites and their waste including uric acid **(E)**, which tends to form MSU crystals in the sodium-rich plasma with time **(F)**. MSU crystals induce endothelial inflammation and NET formation upon contact with circulating neutrophils **(G)**. In healthy conditions, the endothelium is a thin, monolayered, tight structure **(H)**. Intravascular NET autonomously narrow or even clog the post-capillary venules, a process that can be accelerated by the activation of the coagulation cascade **(I)**. The activation of the coagulation cascade, the recruitment of platelets in direct interplay with intravascular NETs, and a plethora of pro-inflammatory mediators cause microvascular occlusion and disrupt the blood–brain barrier, respectively **(J)**.

Malaria is transmitted by mosquitoes and kills 2,000 humans in sub-Saharan Africa each day (1). Most of the victims are children younger than 5 years of age. Out of the five *plasmodium* species causing malaria, *Plasmodium falciparum* is responsible for most of the severe and fatal infections (2). Upon feeding, the female *Anopheles* mosquito inoculates sporozoites that seek out the hosts’ liver within minutes. After a first replication cycle in hepatocytes for 5–8 days, rupture releases the parasite into circulation where erythrocytes are infected. During the second replication cycle *plasmodium* consumes the contents and energy reserves of the erythrocytes, changes the membrane to enable adherence to the vessel walls (3), and produces waste, including crystalline urate (MSU) and hemozoin (4). After 24, 48, or 72 h, depending on the species, the infected erythrocytes synchronously burst and release their content into the circulation and cause first clinical symptoms (Figure 1). The key pathogenic processes that cause severe malaria include rapid increase of infected erythrocytes, destruction of both infected and uninfected erythrocytes, acute inflammation, and microvascular obstruction. The final outcome is a reduced tissue perfusion that leads to downstream events compromising the cellular metabolism (5). We hypothesize that intravascular formation of neutrophil extracellular traps (NETs) contributes to the vasculopathy, driving severe malaria. NETs are web-like structures of highly modified chromatin and antimicrobial peptides released by activated neutrophils (6). In this article, we discuss the evidence supporting the role of NET formation in the pathogenesis of malaria and propose potential therapeutic interventions.

## NETs Cause Vasculopathy in Malaria

NETosis has evolved as an important innate strategy for killing extracellular pathogens (7). The sticky chromatin fibers of NETs immobilize pathogens and inhibit their spread. Uric acid is to be found in infected erythrocytes as a precipitate together with high concentrations of hypoxanthine and the insoluble hemoglobin metabolite hemozoin. During rupture of infected erythrocytes, these compounds are abruptly released into the circulation. In this scenario, insoluble uric acid and hemozoin encounters the immune system as crystalline matter (4) and the former crystallizes in the sodium-rich plasma as MSU (8). These crystals are potent inducers of NETs formation, even in the presence of plasma proteins (8). Hemozoin activates leukocytes through an inflammasome-dependent pathway (9). Indeed, it has been described that NETs with trapped parasites circulate in children infected with *P. falciparum* (10).

In flow chambers perfused with blood, NETs promote fibrin deposition and bind fibronectin and von Willebrand factor, important for platelet adhesion and thrombus propagation (11). NET fibers contain further procoagulant factors. Neutrophil elastase in NETs may proteolytically inactivate tissue factor pathway inhibitor (12). Furthermore, tissue factor can be deposited on extracellular traps, especially those derived from eosinophils, and factor XII is reportedly present and active on NETs (13). The negatively charged DNA in NETs may provide a scaffold for factor XII activation, which is aided by platelets, but the mechanism is still elusive. Fibrin and NETs synergize to augment the microbial defense in a process referred to as immunothrombosis (14). We hypothesize that the formation of NETs and the activation of the coagulation cascade is a double-edged sword. On the one side, they form a barrier on the endothelial surface protecting it from damage by MSU crystals (15) and hemozoin. On the other side, their occurrence limits the bore diameter of capillaries and postcapillary venules. This is prone to restrict perfusion of the end organs. In worst case, intravascular NETs autonomously clog the vessels or initiate disseminated intravascular coagulation (DIC), a condition involving hemorrhage and microthrombosis that synergize in the abolishment of tissue perfusion (Figure 1). The inflammatory mediators released during the coupled processes of NET formation and coagulation cause opening of the neuroimmunological blood–brain barrier and precipitate the clinical disease as, often fatal, cerebral malaria.

The clinical aspects of malaria vary with age, geography, epidemiology, and immunity. In endemic areas, young children and pregnant women are at highest risk to develop severe forms of malaria. Here, we want to interlace NET formation in plasmodium infections as key factor for the development of severe clinical manifestations (Box 1) (2, 16).

Box 1 **Hypotheses for neutrophil extracellular traps (NETs) as pathognomonic factor for severe malaria.**

1. *Impaired consciousness*: Intravascular NET formation and subsequent reduction of capillary bore diameters impair brain perfusion. In addition, toxic metabolites, e.g., ammonium, accumulate due to liver and kidney deficiency.
2. *Prostration*: Lack of perfusion of skeletal muscles due flow restriction in their capillaries. Flooding the patient with cytokines like IL1β.
3. *Multiple convulsions*: NET-driven disseminated intravascular coagulation (DIC) and microthrombi cause multiple focal brain lesions and convulsions.
4. *Acidosis*: NET formation and acidosis are mutually influencing each other. Reduced blood flow causes hypoxia and drop in the plasma pH. However, acidic conditions interfere with NET formation (17).
5. *Hypoglycemia*: Impaired perfusion and high consumption of glucose by *Plasmodium* deprives host tissues from glucose and precipitates end organ damage.
6. *Renal impairment*: Kidney is damaged due to hypoperfusion and glomerular obstruction. Reduced renal uric acid secretion causes hyperuricemia and promotes further NET formation.
7. *Hemorrhages*: NET-driven DIC contributes to the consumption of coagulation factors.
8. *P. falciparum parasitemia* (>10%): Infected as well as ruptured erythrocytes are trapped by intravascular NETs, escaping sequestration in liver and spleen.
9. *Severe malarial anemia, jaundice, pulmonary edema, and shock*: There is no obvious specific contribution of NETs, besides the fact that these symptoms are the consequence of decreased end organ perfusion.

## Discussion

Each year, 240 million people develop symptomatic malaria infections, placing malaria as one of the most serious diseases of mankind. Treatment options in affected areas are challenging and casualties remain high. Here, we propose NET formation as key event determining the severity of disease progression. Parasites within erythrocytes reportedly produce uric acid, which is released as MSU crystals together with crystalline hemozoin synchronously upon rupture of the infected cells. MSU crystals may induce the formation of intravascular NETs, and hemozoin pigments activate neutrophils, immobilizing parasites and crystals at the endothelium. We discussed three possible outcomes (Figure 1). (i) The NETs shield the endothelium, dampening the pro-inflammatory effects of MSU (15) and hemozoin (9). (ii) The NETs and the activation of the coagulation cascade form a barrier on the surface of endothelium, reducing the vessels’ inner diameter and, thereby, the perfusion to virtually all organs. (iii) NETs autonomously, as well as the activation of the coagulation cascade by the negatively charged DNA strands promote DIC. As consequence, hemorrhages and microthrombi may impact microcirculation and promote end organ ischemia. The interplay between intravascular NETs and the coagulation cascade is still elusive. However, the formation of a scaffold in the vasculature by NETs allows the recruitment of coagulation factors, inflammatory mediators, immune cells, and platelets—quite possible being able to disrupt the blood–brain barrier and promote cerebral malaria (18).

Intriguingly, reports indicate a decrease in the counts of circulating granulocytes, monocytes, and lymphocytes in the face of *Plasmodium* infections, especially during gestation. This includes a significant reduction in peripheral neutrophil count (19). We speculate this might be partly due to the formation of NETs in the course of the disease. However, there is no direct evidence correlating the neutrophil count and the release of parasites into the circulation. An abnormal capacity to execute an oxidative burst was described for neutrophils of children suffering from *P. falciparum* malaria (20). The authors show that the reduced oxidative burst capacity in neutrophils occurred after the infection. In a murine model for malaria, this effect was attributed to the induction of the cytoprotective heme oxygenase 1 in neutrophil progenitors of the bone marrow (20). Although NET formation is closely associated with oxidative burst, the aforementioned study did not address NET formation. Therefore, we prompt researchers to include NET formation in their repertoire for functional evaluation of neutrophils. A number of studies argue that antimalarial drugs might influence the capacity of neutrophils to undergo oxidative burst or alter their effector functions (21, 22). Resulting limitation of neutrophils to form NETs might be beneficial in the context of malaria.

The occurrence of anti-neutrophil cytoplasmic antibodies (ANCAs) in 50% of patients with malaria supports the role of NETs in the etiopathogenesis of this, often fatal, disease (23, 24). The detection of these autoantibodies is used in the diagnosis of systemic vasculitides, e.g., granulomatosis with polyangiitis. ANCAs are classified into C- and P-ANCAs according to their antigen specificity for proteinase 3 and myeloperoxidase, respectively. While C- and P-ANCA bind to intact neutrophils, NETs contain only antigens of P-ANCAs (25). Malaria patients primarily develop P-ANCAs (23), supporting the notion that NETs may serve as an autoantigen in this disease.

The involvement of NETs in severe malaria has not been studied in detail. Utilizing NET formation, MSU, and the coagulation cascade as targets of new therapies harbors the potential to reduce the cases with mortality outcomes and alleviate severe forms of malaria infection.

## Author Contributions

SB wrote the manuscript. LM, MH, and TF supervised the project and wrote the manuscript. All the authors read and approved the manuscript.

## Conflict of Interest Statement

The authors declare that the research was conducted in the absence of any commercial or financial relationships that could be construed as a potential conflict of interest.
